# Effects of APOE rs429358, rs7412 and GSTM1/GSTT1 Polymorphism on Plasma and Erythrocyte Antioxidant Parameters and Cognition in Old Chinese Adults

**DOI:** 10.3390/nu7105391

**Published:** 2015-09-24

**Authors:** Linhong Yuan, Jinmeng Liu, Li Dong, Can Cai, Sisi Wang, Bo Wang, Rong Xiao

**Affiliations:** 1 School of Public Health, Nutrition and Food Hygiene Department, Capital Medical University, Beijing 100069, China; ylhmedu@126.com (L.Y.); jinmengliu@ccmu.edu.cn (J.L.); dongli33@126.com (L.D.); caican@sina.com (C.C.); sisiwang@163.com (S.W.); bobowang@ccmu.edu.cn (B.W.); 2 Beijing Key Laboratory of Environmental Toxicology, Capital Medical University, Beijing 100069, China

**Keywords:** cognition, antioxidant biomarkers, polymorphism, the elderly

## Abstract

Apolipoprotein E (APOE) and oxidative damage were correlated with the risk of Alzheimer’s disease (AD). Glutathione *S*-transferase (GST) polymorphism was proved to be associated with body antioxidant capacity and involved in the oxidative damage related chronic diseases. To explore the combined effects of APOE rs429358, rs7412 and GSTM1/T1 polymorphism on antioxidant parameters and cognition in old Chinese adults, a community-based cross-sectional study was carried out in 477 Chinese adults aged from 55 to 75. Information on demography and lifestyle of the participants was collected with a questionnaire. Cognitive function was measured by using a Montreal Cognitive Assessment (MoCA) test. Fasting venous blood samples were collected for APOE rs429358, rs7412 and GSTM1/T1 genotyping, and parameter measurement. No association of APOE rs7412, rs429358 and GSTM1/T1 polymorphisms with cognition was detected in the old Chinese adults. APOE rs429358, rs7412 polymorphism was mainly associated with plasma α-tocopherol, low density lipoprotein cholesterol (LDL-C) and plasma total antioxidant capacity (T-AOC) levels (*p* < 0.05). Interaction of APOE rs429358 and GSTT1 genotype on the plasma triglyceride (TG) level and erythrocyte catalase (CAT) and GST enzyme activities were detected (*p* < 0.05). The subjects with APOE rs429358 T/C + C/C and GSTT1− genotype were found to have the highest plasma TG level, erythrocyte CAT enzyme activity, and the lowest GST enzyme activity compared to subjects with other genotypes (*p* < 0.05). Lowest erythrocyte CAT enzyme activity and highest glutathione peroxidase (GSH-Px) enzyme activity were detected in the subjects with APOE rs7412 T/C + T/T and GSTM1+ genotype as compared with subjects with other genotypes. The levels of plasma and erythrocyte antioxidant parameters were APOE genotype associated. GSTM1 or GSTT1 genotype modified the influence of APOE rs7412, rs429358 polymorphism on antioxidant parameters.

## 1. Introduction

Alzheimer’s disease (AD) is the most common progressive neurodegenerative disorder leading to dementia. It has been estimated that at least 10% of people who are 65 years or older have some forms of cognitive impairment [1]. However, up to date, the pathogenesis of AD is still unclear; and there is still no efficiency method available for the prevention and treatment of AD. Therefore, biomarkers that predict future risks are of critical importance to design targeted prevention of cognitive decline and AD.

Brain is particularly vulnerable to oxidative stress as a result of the relatively low levels of antioxidants and high levels of omega-3 polyunsaturated fatty acids (such as docosahexenoic acid) [2]. Increasing published documents implicate the contribution of oxidative damage to the pathogenesis of AD [3,4]. Post-mortem and *in vivo* studies have demonstrated an accumulation of products of free radical damage in the central nervous system and in the peripheral tissues of subjects with AD or mild cognitive impairment [5,6,7]. Also, this situation is combined with a reduction of most enzymatic antioxidants defenses, like glutathione peroxidase (GPX), glutathione *S*-transferase (GST) and superoxide dismutase (SOD) [8] accompanied with elevated markers of lipid peroxidation, like malondialdehyde (MDA) [9,10]. These data further proved the imbalance between pro-oxidants and antioxidants involved in the pathogenesis of AD. Thus, oxidative damage might represent a potential therapeutic target for slowing the onset and progression of AD [11].

Apolipoprotein E (APOE) has a major physiological role in the regulation of overall lipid and lipoprotein homeostasis. Additionally, APOE plays an important role in neuronal repair. APOE ε4 allele, derived from the combination of polymorphisms rs429358 and rs7412, is the biomarker with the greatest known influence on the risk of developing AD [12]. In addition to conferring a higher risk of AD, the presence of APOE ε4 allele can significantly affect cognitive performance in non-demented subjects [13]. Moreover, APOE has been shown to possess antioxidant and anti-inflammatory properties in a genotype-dependent manner. The APOE ε4 allele was reported to be associated with a relatively higher oxidative stress and a higher pro-inflammatory state [14,15,16].

GSTs belong to a large family of different enzymes that catalyze the *S*-conjugation of glutathione with a wide variety of electrophilic compounds, including reactive oxygen species (ROS) [17]. Reduced GST activity has been reported in multiple brain regions and in ventricular cerebrospinal fluid in short postmortem interval AD patients [18]. The GSTM1 and GSTT1 classes of enzymes have frequently occurring phenotypes that derive from deletion of the respective genes, called “null phenotypes” [19,20]. Approximately 35%–66% of the Chinese population is homozygous for a deletion of the GSTM1 gene; and 50%–66% are homozygous for a deletion of the GSTT1 gene [21]. The role of the GST polymorphisms in carcinogenesis and drug resistance has been extensively explored. However, limited studies have focused on the effect of GST polymorphism on neurological diseases.

The oxidative damage and APOE genotype are important factors involved in the pathogenesis of AD. Recently, genotype–environment interaction was proven to cause various degree of association between gene (such as APOE) polymorphism and body parameters with AD susceptibility in different populations. Besides, polymorphism of GSTM1 and GSTT1 was indicated contributing to the risk of oxidative damage related chronic diseases. Therefore, we speculate that the combine of APOE and GSTM1/GSTT1 polymorphism might influence body antioxidant related biomarkers, and finally it might affect cognition in the elderly. Due to the much lower frequencies of APOE ε4 alleles in Asian populations compared to that in the Northern European population, therefore, in the present study, we evaluated the influence of APOE rs429358, rs7412 polymorphisms, respectively, on plasma, erythrocyte antioxidant parameters and cognition in old Chinese adults. We also include GSTM1 and GSTT1 genetic polymorphisms into the relationship exploration between APOE genotype and body antioxidant parameters, aiming to uncover the relationship among APOE, GSTM1/GSTT1 polymorphism and cognition and body antioxidant capacity in the older Chinese adults.

## 2. Materials and Method

### 2.1. Participants

The study was a community-based cross-sectional study, and the study protocol was approved by the Human Ethics Committee of the Capital Medical University (No. 2012SY23). The procedures followed the ethical standards of the Helsinki Declaration of 1975. A total of 504 community dwellers aged 55–75 were randomly recruited by advertisements and phone dialing by the nurses in the service center from Nanyuan Community, Beijing, China. Medical doctors interviewed the participants face-to-face in Nanyuan Community Health Service Center. Criteria for exclusion of the subjects were conditions known to affect biological variables of oxidative stress or cognitive function (e.g., inflammatory diseases, recent history of heart or respiratory failure, chronic liver or renal failure, malignant tumors, a recent history of alcohol abuse, history of cerebral apoplexy, history of cerebral infarction, antioxidant supplementary). The subjects with AD or Parkinson’s disease (PD), or those not being able to complete the cognition test or taking long-term, frequent anti-depressants and central nervous system acting medications were also excluded from the study. Written informed consent was obtained from all participants.

### 2.2. Cognitive Tests

Cognitive function was measured by using Montreal Cognitive Assessment (MoCA), which assessed several cognitive domains including the short-term memory recall ability, visuospatial abilities, executive functions, phonemic fluency ability, verbal abstraction ability, attention, concentration and working memory, language and orientation. This assessment was validated in the detection of mild cognitive impairment and early Alzheimer’s disease [22], and has been widely used in other large-scale studies on cognitive function in the elderly previously [23]. The test was carried out by trained investigators in the community health service center.

### 2.3. Sociodemographic Variables and Anthropometric Measurements

Anthropometric measures (height and weight) were made by the nurse in the community medical service center. BMI was calculated as weight (kg)/height (m^2^). Information on demographic characteristics (for example, gender, age, education and marital status), lifestyle factors (for example, smoking and physical activity), medical history of chronic diseases and dietary supplements was collected by using a self-administered questionnaire. Educational level was assessed as the highest level reached and classified into four categories (illiterate, primary school, junior high school, and > high school). Smoking status was defined as being a non-smoker or current smoker. Physical activity level was classified into three categories: inactive (no physical activity), moderately active (1–3 times per week), and active (everyday).

Participation in cognitive activity refers to time expended on reading books and computer use, which was classified into three categories: <30 min per day, 30–60 min per day, and >60 min per day.

### 2.4. DNA Isolation and Genotyping

Peripheral blood samples (6 mL venipuncture) were collected in EDTA tube and stored at −80 °C. DNA was extracted from frozen peripheral blood using the Wizare genomic DNA purification kit (Promega, Madison, WI, USA). GSTT1 and GSTM1 genotypes were determined by multiple polymerase chain reaction (PCR) method using primers and reaction profiles as described by Zhong [24,25] respectively. The APOE genotypes were determined by PCR amplification and Restricted Fragment Length Polymorphism (RFLP) analysis according to the method described by Hixson [26]. The specific primers used for GSTM1/GSTT1 and APOE genotyping are: GSTM1 primers: forward, 5’-GAA CTC CCT GAA AAG CTA AAG C-3’; reverse, 5’-GTT GGG CTC AAA TAT ACG GTG G-3’; GSTT1: forward, 5’-TTC CTT ACT GGT CCT CAC ATC TC-3’; reverse, 5’-TCA CCG GAT CAT GGC CAG CA-3’; β-globin: forward, 5’-GAA GAG CCA AGG ACA GGT AC-3’; reverse, 5’-CAA CTT CAT CCA CGT TCA CC-3’; APOE primers: forward, 5’-GGC ACG GCT GTC CAA GGA-3’; reverse, 5’-GCC CCG GCC TGG TAC ACT GCC-3’. For the purpose of quality control of the genotyping, 20% of DNA samples were genotyped again by different operators.

### 2.5. Plasma and Erythrocyte Parameter Measurement

For biochemical analysis, blood samples were collected into lithium heparin tubes and centrifuged at 480 g for 10 min at 4 °C, and then the plasma was stored at −20 °C until further analysis. An ILAB600 clinical chemistry analyzer (Instrumentation Laboratory, Lexington, WI, USA) was used to determine plasma glucose (Glu), triglyceride (TG), total cholesterol (TC). High density lipoprotein cholesterol (HDL-C) was measured by using a commercially available assay from Instrumentation Laboratory (Lexington, WI, USA). Low density lipoprotein cholesterol (LDL-C) was calculated using the Friedewald formula [27]. All samples for each participant were analyzed within a single batch, and the inter-assay coefficients of variation (CV) were less than 5%.

For the antioxidant parameter analysis, blood samples were centrifuged at 480 g for 20 min. The plasma and erythrocyte were separated, and transferred to storage tubes and frozen at −80 °C for further analysis. Plasma total antioxidant capacity (T-AOC), MDA level, erythrocyte catalase (CAT) activity, SOD activity, total GST enzyme activity, glutathione peroxidase (GSH-Px) activity, glutathione reductase (GR) activity were measured by using commercial assay kits (Nanjing Jiancheng Bioengineering Institute, Nanjing, China) according to the manufacturer’s instruction. Two independent measurements were performed for each sample. T-AOC was expressed as U/mL; MDA was expressed as nmol/L. Erythrocyte enzyme activity was normalized per microgram of hemoglobin. The intra- and inter-assay CVs for all measurements were 5%.

### 2.6. Measurement of Plasma Vitamin Level

After extraction with ethanol and hexane, plasma retinol and α-tocopherol were determined by using high-performance liquid chromatography (HPLC) (Gilson, Middleton, WI, USA) with UV detection at 280 nm with a Waters Symmetry C8 column (150 mm × 4.6 μm) according to the method described by Nierenberg [28]. All the extraction and solvents were purchased from Sigma-Aldrich (St. Louis, MO, USA). Plasma folate concentration was measured by using the assay kit purchased from Bayer AG (Leverkusen, Germany) according to the manufacturer’s instruction. Two independent measurements were performed for each sample.

### 2.7. Statistical Analyses

Data was analyzed with the software SPSS 19.0 (Chicago, IL, USA). All continuous variables are presented as means ± standard deviation (SD), or means (95% confidence intervals). Age, gender, living status, education level, smoking, usage of antioxidant supplement, physical activity and cognitive activity were presented as category variables. Participants were classified according to categories of APOE and/or GST genotypes. General linear model (GLM) was used to compare the means of the detected parameters between the groups. Some potential confounding factors including gender (male, female), age (year), BMI, smoking (yes or no), and physical activity were adjusted during the data analysis of plasma Glu, TG, TC, LDL-C and HDL-C. For erythrocytes antioxidant biomarkers, plasma folate, retinol and α-tocopherol data analysis, factors including gender, age, BMI, smoking, and antioxidant supplementation were adjusted. For comparing cognition (MoCA score), factors including gender, age, BMI, physical activity, education level, living condition, cognitive activity and antioxidant supplementation (yes or no) were adjusted. *p* < 0.05 was considered to be statistically significant.

## 3. Results

### 3.1. Demographics of the Participants

Initially, a total of 504 older adults participated in the present study. Twenty-seven individuals were excluded due to uncompleted questionnaires, unsuccessful biological specimen sampling or unsuccessful genotyping. After eliminating missing data, 477 subjects were included in the statistical analyses. The demographic characteristics of the participants were presented in Table 1. As shown in Table 1, the mean age of the participants was 63.23 ± 5.35; 35.22% of the subjects were male; the mean BMI of the subjects was 25.221 ± 3.406; majority (89.4%) of them had an education level above junior high school. The subjects who live alone accounted for 5.03% of all participants. In total, 17.61% of the subjects had the habit of smoking. Among the 477 subjects, only three subjects were of the APOE rs429358 C/C genotype (accounting for 0.63% of all subjects); only three subjects were of the APOE rs7412 T/T genotype (accounting for 0.42% of all subjects). Therefore, during the data analysis, the carriers of one or two copies of the C allele were pooled for APOE rs429358; and the carriers of one or two copies of the T allele were pooled for APOE rs7412. Among the 477 subjects, 207 (43.40% of all subjects) were of the GSTM1+ genotype, and 241 (50.52% of all the subjects) were of the GSTT1+ genotype.

**Table 1:** Demographic characteristics of the participants.

Characteristics	Total (*n* = 477)
***Demographic variables***	
Age (mean ± S.E.)	63.23 ± 5.349
Male gender, *n* (%)	168 (35.220)
BMI (mean ± S.E.)	25.221 ± 3.406
Living along, *n* (%)	24 (5.031)
Smoking, *n* (%)	84 (17.610)
Education	
Illiterate, *n* (%)	6 (1.258)
Primary school, *n* (%)	45 (9.434)
Junior high school, *n* (%)	228 (47.799)
High school and above, *n* (%)	194 (40.671)
Antioxidant supplement, *n* (%)	33 (6.918)
***APOE SNPs***	
rs 429358	
T/T, *n* (%)	401 (84.067)
C/T, *n* (%)	73 (15.304)
C/C, *n* (%)	3 (0.629)
rs7412	
T/T, *n* (%)	2 (0.419)
C/T, *n* (%)	68 (14.256)
C/C, *n* (%)	407 (85.325)
***GST genotype***	
GSTM1+, *n* (%)	207 (43.396)
GSTT1+, *n* (%)	241 (50.524)

Abbreviations: BMI, body mass index; APOE, apolipoprotein E; GST, glutathione *S*-transferase; SNPs: Single nucleotide polymorphisms; S.E., standard error of the mean.

### 3.2. Plasma, Erythrocyte Parameters and Cognition according to APOE rs429358

As shown in Table 2, APOE rs429358 polymorphism has no effect on plasma lipid profile. Subjects with APOE rs429358 T/T genotype seem to have lower plasma MDA and T-AOC levels than the subjects with APOE rs429358 T/C + C/C genotype. Comparing with APOE rs429358 T/C + C/C genotype carriers, subjects with APOE rs429358 T/T genotype have lower plasma α-tocopherol level (*p* < 0.05). APOE rs429358 polymorphism has no effect on plasma folate and retinol levels. The erythrocyte antioxidant enzyme activities and cognition (MoCA score) of the detected subjects were not influenced by APOE rs429358 polymorphism.

**Table 2:** Plasma, erythrocyte parameters and cognition according to APOE rs429358.

Biomarkers and Cognition	rs429358		*p* Value
	T/T (*n* = 363)	T/C + C/C (*n* = 69)	
***Plasma***			
Glu (mmol/L)	5.471 (5.288, 5.655)	5.183 (4.761, 5.606)	0.220
TG (mmol/L)	1.526 (1.435, 1.617)	1.730 (1.521, 1.939)	0.080
TC (mmol/L)	4.813 (4.718, 4.907)	4.990 (4.772, 5.208)	0.144
LDL-C (mmol/L)	3.238 (3.147, 3.329)	3.378 (3.168, 3.587)	0.231
HDL-C (mmol/L)	1.331 (1.295, 1.367)	1.346 (1.263, 1.429)	0.759
MDA (nmol/L)	6.768 (6.172, 7.364)	7.846 (6.472, 9.220)	0.158
T-AOC (activity U/L)	11.626 (11.200, 12.053)	12.083 (11.099, 13.066)	0.404
Retinol (μg/mL)	0.727 (0.713, 0.742)	0.709 (0.677, 0.724)	0.314
α-tocopherol (μg/mL)	10.372 (10.066, 10.678)	11.328 (10.627, 12.029)	0.014
Folate (μg/L)	6.230 (5.811, 6.649)	6.241 (5.281, 7.201)	0.984
***Erythrocyte***			
SOD (U/g Hb)	31.408 (30.951, 31.864)	31.926 (30.878, 32.974)	0.373
CAT (U/g Hb)	2.369 (2.330, 2.408)	2.316 (2.228, 2.405)	0.284
GSH-PX (U/g Hb)	25.844 (25.075, 26.612)	26.286 (24.523, 28.050)	0.651
GST (U/g Hb)	0.342 (0.327, 0.358)	0.318 (0.283, 0.353)	0.203
GR (U/g Hb)	0.552 (0.534, 0.570)	0.551 (0.509, 0.593)	0.961
***Cognition***			
Moca score	26.353 (26.060, 26.646)	26.597 (25.927, 27.266)	0.513

General linear model used. Data are presented as the mean (95% CI). Glu: glucose; TG: triglyceride; TC: total cholesterol; LDL-C: low density lipoprotein cholesterol; HDL-C: high density lipoprotein cholesterol; MDA: malondialdehyde; T-AOC: total antioxidant capacity; SOD: superoxide dismutase; CAT: catalase; GSH-Px: glutathione peroxidase; GST: glutathione *S*-transferase; GR: glutathione reductase; MoCA, Montreal Cognitive Assessment. For plasma Glu, TG, TC, LDL-C and HDL-C data analysis, factors including gender, age, BMI, smoking and physical activity were adjusted. For erythrocyte antioxidant biomarkers and plasma folate, retinol and α-tocopherol data analysis, factors including gender, age, BMI, smoking and antioxidant supplementation were adjusted. For cognition (MoCA score) analysis, factors including gender, age, BMI, physical activity, education level, living condition, reading time per day and antioxidant supplementation were adjusted. *p* value < 0.05 was considered to be significant.

### 3.3. Plasma, Erythrocyte Parameters and Cognition According to APOE rs7412

In contrast to individuals with APOE rs7412 C/C genotype, subjects with APOE rs7412 T/C + T/T genotype have a lower plasma LDL-C level (*p* < 0.01) and higher T-AOC (*p* < 0.05). Plasma folate, α-tocopherol, retinol levels, erythrocytes antioxidant enzyme activities and cognition were not associated with APOE rs7412 polymorphism (Table 3).

**Table 3:** Plasma, erythrocytes parameters and cognition according to APOE rs7412.

Biomarkers and Cognition	rs7412		*p* Value
	T/C + T/T (*n* = 64)	C/C (*n* = 368)	
***Plasma***			
Glu (mmol/L)	5.623 (5.197, 6.049)	5.394 (5.214, 5.575)	0.332
TG (mmol/L)	1.654 (1.432, 1.876)	1.545 (1.451, 1.639)	0.375
TC (mmol/L)	4.772 (4.551, 4.993)	4.846 (4.753, 4.940)	0.543
LDL-C (mmol/L)	2.985 (2.775, 3.195)	3.298 (3.210, 3.387)	0.007
HDL-C (mmol/L)	1.376 (1.292, 1.459)	1.327 (1.291, 1.362)	0.290
MDA (nmol/L)	6.552 (5.172, 7.932)	6.960 (6.376, 7.543)	0.593
T-AOC (activity U/L)	10.488 (9.436, 11.459)	11.773 (11.356, 12.190)	0.018
Retinol (μg/mL)	0.728 (0.695, 0.762)	0.724 ( 0.709, 0.738)	0.800
α-tocopherol (μg/mL)	10.570 (9.848, 11.293)	10.517 (10.210, 10.825)	0.895
Folate (μg/L)	5.787 (4.805, 6.768)	6.313 (5.895, 6.730)	0.334
***Erythrocyte***			
SOD (U/g Hb)	31.676 (30.550, 32.803)	31.444 (30.980, 31.908)	0.708
CAT (U/g Hb)	2.322 (2.228, 2.416)	2.365 (2.326, 2.404)	0.409
GSH-PX (U/g Hb)	26.344 (24.455, 28.233)	25.905 (25.127, 26.683)	0.673
GST (U/g Hb)	0.352 (0.315, 0.389)	0.335 (0.320, 0.351)	0.408
GR (U/g Hb)	0.593 (0.548, 0.637)	0.547 (0.529, 0.566)	0.068
***Cognition***			
Moca score	25.975 (25.284, 26.667)	26.467 (26.177, 26.757)	0.199

General linear model used. Data are presented as the mean (95% CI). For plasma Glu, TG, TC, LDL-C and HDL-C data analysis, factors including gender, age, BMI, smoking, physical activity were adjusted. For erythrocytes antioxidant biomarkers and plasma folate, retinol and α-tocopherol data analysis, factors including gender, age, BMI, smoking and antioxidant supplementation were adjusted. For cognition (MoCA score) analysis, factors including gender, age, BMI, physical activity, education level, living condition, reading time per day and antioxidant supplementation were adjusted. *p* value < 0.05 was considered as significance.

### 3.4. Plasma, Erythrocytes Parameters and Cognition according to APOE rs429358 and GSTM1/T1 Polymorphism

The combined effects of APOE rs429358 and GSTM1 polymorphism on plasma, erythrocytes parameters and cognition were presented in Table 4. No relationship between APOE rs429358 and GSTM1 polymorphism and detected parameters and cognition was detected in old Chinese adults (*p* > 0.05).

As shown in Table 5, combined effects of APOE rs429358 and GSTT1 polymorphism on plasma TG level, erythrocytes CAT and GST enzyme activities (*p* < 0.05) were detected. Comparing with the subjects with other genotypes, the subjects with APOE rs429358 T/T and GSTT1+ genotype have the lowest plasma TG levels, and the subjects with APOE rs429358 T/C + C/C and GSTT1− genotype have the highest plasma TG levels. The subjects with APOE rs429358 T/C + C/C and GSTT1+ genotype have the lowest erythrocyte CAT enzyme activity. Furthermore, the subjects with APOE rs429358 T/C + C/C and GSTT1− genotype have the lowest erythrocyte GST activity comparing with subjects with other genotypes. APOE rs429358 and GSTT1 polymorphism have no effect on cognition and plasma retinol, folate and α-tocopherol levels in the old adults (*p* > 0.05).

### 3.5. Plasma, Erythrocytes Parameters and Cognition according to APOE rs7412 and GSTM1/T1 Polymorphism

No association of APOE rs7412 and GSTM1 polymorphism with plasma lipid profile, MDA, T-AOC, retinol, folate and α-tocopherol levels was found in the current study (*p* > 0.05) (Table 6). Carriers of APOE rs7412 T/C + T/T and GSTM1+ genotype have the lowest erythrocyte CAT enzyme activity and the highest erythrocyte GSH-Px enzyme activity (*p* < 0.05) as comparing with subjects with other genotypes (*p* < 0.01). No difference was detected on the cognition (MoCA score) among the subjects (*p* > 0.05) (Table 6). As shown in Table 7, APOE rs7412 and GSTT1 polymorphism have no effect on the detected parameters and cognition (MoCA score) in old Chinese adults (*p* > 0.05).

**Table 4:** Plasma, erythrocytes parameters and cognition according to APOE rs429358 and GSTM1 genotype.

Biomarkers and Cognition	rs429358				*p* Value
	T/T (*n* = 363)		T/C + C/C (*n* = 69)		
	GSTM1+ (*n* = 165)	GSTM1− (*n* = 198)	GSTM1+ (*n* = 36)	GSTM1− (*n* = 33)	
***Plasma***					
Glu (mmol/L)	5.604 (5.327, 5.881)	5.486 (5.235, 5.736)	4.974 (4.384, 5.564)	5.423 (4.805, 6.041)	0.233
TG (mmol/L)	1.573 (1.436, 1.710)	1.494 (1.370, 1.618)	1.843 (1.551, 2.135)	1.604 (1.298, 1.909)	0.494
TC (mmol/L)	4.886 (4.748, 5.023)	4.738 (4.614, 4.863)	5.049 (4.756, 5.343)	4.948 (4.641, 5.255)	0.845
LDL-C (mmol/L)	3.315 (3.183, 3.447)	3.161 (3.042, 3.281)	3.386 (3.104, 3.667)	3.396 (3.101, 3.690)	0.470
HDL-C (mmol/L)	1.331 (1.279, 1.382)	1.325 (1.278, 1.372)	1.337 (1.227, 1.447)	1.352 (1.237, 1.467)	0.814
MDA (nmol/L)	7.226 (6.343, 8.108)	6.397 (5.591, 7.202)	8.490 (6.601, 10.378)	7.079 (5.093, 9.066)	0.703
T-AOC (activity U/L)	11.538 (10.905, 12.171)	11.700 (11.123, 12.278)	12.540 (11.185, 13.894)	11.579 (10.154, 13.004)	0.304
Retinol (μg/mL)	0.719 (0.698, 0.740)	0.734 (0.715, 0.753)	0.713 (0.667, 0.759)	0.705 (0.658, 0.752)	0.532
α-tocopherol (μg/mL)	10.503 (10.044, 10.961)	10.267 (9.856, 10.677)	12.000 (11.023, 12.977)	10.622 (9.617, 11.627)	0.144
Folate (μg/L)	5.960 (5.334, 6.586)	6.451 (5.891, 7.012)	6.674 (5.341, 8.008)	5.758 (4.386, 7.130)	0.187
***Erythrocyte***					
SOD (U/g Hb)	31.608 (30.897, 32.318)	31.263 (30.615, 31.912)	32.196 (30.676, 33.717)	31.757 (30.157, 33.357)	0.938
CAT (U/g Hb)	2.363 (2.304, 2.423)	2.359 (2.305, 2.414)	2.285 (2.157, 2.412)	2.320 (2.186, 2.454)	0.701
GSH-PX (U/g Hb)	26.201 (25.006, 27.396)	25.745 (24.654, 26.835)	27.176 (24.619, 29.733)	25.765 (23.075, 28.456)	0.644
GST (U/g Hb)	0.333 (0.309, 0.356)	0.351 (0.329, 0.372)	0.343 (0.293, 0.393)	0.291 (0.238, 0.344)	0.083
GR (U/g Hb)	0.567 (0.539, 0.596)	0.545 (0.519, 0.570)	0.543 (0.482, 0.603)	0.581 (0.517, 0.644)	0.214
***Cognition***					
Moca score	26.250 (25.813, 26.686)	26.433 (26.037, 26.830)	26.901 (25.974, 27.828)	26.296 (25.328, 27.263)	0.290

General linear model used. Data are presented as the mean (95% CI). For plasma Glu, TG, TC, LDL-C, and HDL-C data analysis, factors including gender, age, BMI, smoking, physical activity were adjusted. For erythrocytes antioxidant biomarkers and plasma folate, retinol, and α-tocopherol data analysis, factors including gender, age, BMI, smoking, and antioxidant supplementation were adjusted. For cognition (MoCA score) analysis, factors including gender, age, BMI, physical activity, education level, living condition, reading time per day, and antioxidant supplementation were adjusted. *p* value < 0.05 was considered as significant.

**Table 5:** Plasma, erythrocytes parameters and cognition according to APOE rs429358 and GSTT1 genotype.

Biomarkers and Cognition	rs429358				*p* Value
	T/T (*n* = 363)		T/C + C/C (*n* = 69)		
	GSTT1+ (*n* = 188)	GSTT1− (*n* = 175)	GSTT1+ (*n* = 39)	GSTT1− (*n* = 30)	
***Plasma***					
Glu (mmol/L)	5.815 (5.558, 6.071)	5.246 (4.981, 5.510)	5.255 (4.703, 5.806)	5.094 (4.434, 5.754)	0.393
TG (mmol/L)	1.535 (1.408, 1.663)	1.523 (1.392, 1.655)	1.523 (1.248, 1.797)	2.024 (1.695, 2.352)	0.031
TC (mmol/L)	4.761 (4.632, 4.890)	4.851 (4.718, 4.984)	4.983 (4.705, 5.260)	5.027 (4.696, 5.359)	0.849
LDL-C (mmol/L)	3.187 (3.063, 3.310)	3.278 (3.150, 3.405)	3.426 (3.160, 3.692)	3.339 (3.021, 3.658)	0.439
HDL-C (mmol/L)	1.313 (1.264, 1.361)	1.343 (1.294, 1.393)	1.323 (1.220, 1.427)	1.373 (1.249, 1.497)	0.827
MDA (nmol/L)	6.318 (5.491, 7.145)	7.260 (6.403, 8.117)	7.723 (5.898, 9.547)	7.951 (5.877, 10.026)	0.642
T-AOC (activity U/L)	11.241 (10.649, 11.832)	12.040 (11.427, 12.652)	12.056 (10.752, 13.360)	12.122 (10.639, 13.605)	0.504
Retinol (μg/mL)	0.736 (0.715, 0.756)	0.720 (0.700, 0.739)	0.697 (0.646, 0.748)	0.718 (0.675, 0.761)	0.316
α-tocopherol (μg/mL)	10.696 (10.256, 11.136)	10.071 (9.648, 10.494)	12.180 (11.094, 13.265)	10.730 (9.817, 11.643)	0.295
Folate (μg/L)	6.153 (5.549, 6.757)	6.306 (5.725, 6.887)	6.086 (4.595, 7.578)	6.329 (5.075, 7.584)	0.934
***Erythrocyte***					
SOD (U/g Hb)	31.342 (30.677, 32.007)	31.504 (30.814, 32.193)	31.494 (30.027, 32.962)	32.627 (30.958, 34.295)	0.432
CAT (U/g Hb)	2.377 (2.322, 2.432)	2.345 (2.287, 2.402)	2.213 (2.091, 2.336)	2.451 (2.276, 2.554)	0.023
GSH-PX (U/g Hb)	25.157 (24.042, 26.272)	26.803 (25.648, 27.958)	26.785 (24.325, 29.245)	26.153 (23.356, 28.950)	0.271
GST (U/g Hb)	0.323 (0.294, 0.390)	0.363 (0.341, 0.386)	0.342 (0.294, 0.390)	0.288 (0.233, 0.342)	0.020
GR (U/g Hb)	0.546 (0.519, 0.527)	0.565 (0.537, 0.592)	0.551 (0.493, 0.610)	0.573 (0.506, 0.639)	0.963
***Cognition***					
Moca score	26.344 (25.936, 26.752)	26.357 (25.936, 26.779)	26.731 (25.858, 27.605)	26.439 (25.392, 27.486)	0.686

General linear model used. Data are presented as the mean (95% CI). For plasma Glu, TG, TC, LDL-C, and HDL-C data analysis, factors including gender, age, BMI, smoking, physical activity were adjusted. For erythrocytes antioxidant biomarkers and plasma folate, retinol, and α-tocopherol data analysis, factors including gender, age, BMI, smoking, and antioxidant supplementation were adjusted. For cognition (MoCA score) analysis, factors including gender, age, BMI, physical activity, education level, living condition, reading time per day, and antioxidant supplementation were adjusted. *p* value < 0.05 was considered as significant.

**Table 6:** Plasma, erythrocytes parameters and cognition according to APOE rs7412 and GSTM1 genotype.

Biomarkers and Cognition	rs7412				*p* Value
	T/C + T/T (*n* = 64)		C/C (*n* = 368)		
	GSTM1+ (*n* = 26)	GSTM1− (*n* = 38)	GSTM1+ (*n* = 175)	GSTM1− (*n* = 193)	
***Plasma***					
Glu (mmol/L)	5.411 (4.735, 6.087)	5.994 (5.424, 6.564)	5.504 (5.234, 5.773)	5.373 (5.118, 5.627)	0.145
TG (mmol/L)	1.662 (1.327, 1.998)	1.655 (1.373, 1.938)	1.615 (1.482, 1.749)	1.480 (1.354, 1.607)	0.597
TC (mmol/L)	4.954 (4.617, 5.291)	4.662 (4.378, 4.946)	4.909 (4.775, 5.044)	4.789 (4.662, 4.916)	0.481
LDL-C (mmol/L)	3.144 (2.824, 3.465)	2.899 (2.628, 3.169)	3.357 (3.229, 3.485)	3.254 (3.134, 3.375)	0.537
HDL-C (mmol/L)	1.386 (1.261, 1.512)	1.364 (1.258, 1.470)	1.323 (1.273, 1.373)	1.322 (1.274, 1.369)	0.818
MDA (nmol/L)	7.381 (5.151, 9.611)	6.018 (4.170, 7.867)	7.470 (6.611, 8.329)	6.581 (5.763, 7.400)	0.766
T-AOC (activity U/L)	11.289 (9.706, 12.874)	9.962 (8.649, 11.275)	11.780 (11.169, 12.390)	12.023 (11.442, 12.605)	0.166
Retinol (μg/mL)	0.715 (0.662, 0.767)	0.737 (0.693, 0.780)	0.718 (0.698, 0.739)	0.728 (0.709, 0.748)	0.751
α-tocopherol (μg/mL)	10.769 (9.635, 11.903)	10.421 (9.483, 11.360)	10.774 (10.323, 11.225)	10.297 (9.875, 10.718)	0.874
Folate (μg/L)	5.285 (3.749, 6.820)	6.202 (4.932, 7.472)	6.216 (5.605, 6.826)	6.383 (5.812, 6.953)	0.496
***Erythrocyte***					
SOD (U/g Hb)	32.212 (30.416, 34.007)	31.362 (29.874, 32.850)	31.639 (30.947, 32.330)	31.329 (30.670, 31.988)	0.674
CAT (U/g Hb)	2.149 (2.000, 2.298)	2.436 (2.313, 2.559)	2.379 (2.322, 2.437)	2.337 (2.283, 2.392)	0.002
GSH-PX (U/g Hb)	29.122 (26.121, 32.123)	24.438 (21.951, 26.925)	25.961 (24.806, 27.117)	26.011 (24.910, 27.113)	0.028
GST (U/g Hb)	0.351 (0.291, 0.410)	0.358 (0.309, 0.408)	0.332 (0.309, 0.355)	0.339 (0.317, 0.361)	0.991
GR (U/g Hb)	0.615 (0.543, 0.686)	0.584 (0.525, 0.643)	0.555 (0.528, 0.582)	0.543 (0.517, 0.569)	0.713
***Cognition***					
Moca Score	26.260 (25.197, 27.322)	25.759 (24.843, 26.675)	26.384 (25.959, 26.809)	26.540 (26.140, 26.941)	0.399

General linear model used. Data are presented as the mean (95% CI). For plasma Glu, TG, TC, LDL-C, and HDL-C data analysis, factors including gender, age, BMI, smoking, physical activity were adjusted. For erythrocytes antioxidant biomarkers and plasma folate, retinol, and α-tocopherol data analysis, factors including gender, age, BMI, smoking, and antioxidant supplementation were adjusted. For cognition (MoCA score) analysis, factors including gender, age, BMI, physical activity, education level, living condition, reading time per day, and antioxidant supplementation were adjusted. *p* value < 0.05 was considered as significant.

**Table 7:** Plasma, erythrocytes parameters and cognition according to APOE SNP rs7412 and GSTT1 genotype.

Biomarkers and Cognition	rs7412				*p* Value
	T/C + T/T (*n* = 64)		C/C (*n* = 368)		
	GSTT1+ (*n* = 38)	GSTT1− (*n* = 26)	GSTT1+ (*n* = 189)	GSTT1− (*n* = 179)	
***Plasma***					
Glu (mmol/L)	6.105 (5.546, 6.664)	5.220 (4.535, 5.905)	5.633 (5.377, 5.889)	5.225 (4.962, 5.488)	0.329
TG (mmol/L)	1.756 (1.477, 2.036)	1.510 (1.168, 1.852)	1.486 (1.358, 1.614)	1.605 (1.473, 1.737)	0.135
TC (mmol/L)	4.746 (4.464, 5.027)	4.839 (4.494, 5.184)	4.812 (4.683, 4.941)	4.881 (4.749, 5.014)	0.921
LDL-C (mmol/L)	3.006 (2.738, 3.274)	2.991 (2.663, 3.319)	3.277 (3.154, 3.399)	3.330 (3.204, 3.456)	0.768
HDL-C (mmol/L)	1.323 (1.218, 1.428)	1.449 (1.321, 1.577)	1.313 (1.265, 1.361)	1.332 (1.283, 1.382)	0.246
MDA (nmol/L)	5.951 (4.103, 7.798)	7.473 (5.235, 9.712)	6.665 (5.838, 7.492)	7.362 (6.511, 8.214)	0.606
T-AOC (activity U/L)	10.290 (8.980, 11.601)	10.807 (9.219, 12.395)	11.596 (11.010, 12.183)	12.236 (11.632, 12.840)	0.913
Retinol (μg/mL)	0.730 (0.686, 0.773)	0.725, 0.673, 0.778)	0.717 (0.697, 0.737)	0.731 (0.710, 0.752)	0.630
α-tocopherol (μg/mL)	10.403 (9.469, 11.337)	10.786 (9.654, 11.917)	10.145 (9.719, 10.570)	10.923 (10.481, 11.365)	0.625
Folate (μg/L)	5.827 (4.556, 7.098)	5.835 (4.296, 7.375)	6.410 (5.831, 6.990)	6.191 (5.589, 6.793)	0.837
***Erythrocyte***					
SOD (U/g Hb)	32.104 (30.620, 33.589)	31.117 (29.318, 32.916)	31.219 (30.554, 31.883)	31.749 (31.065, 32.434)	0.238
CAT (U/g Hb)	2.378 (2.254, 2.502)	2.232 (2.082, 2.382)	2.342 (2.287, 2.398)	2.373 (2.316, 2.431)	0.100
GSH-PX (U/g Hb)	26.569 (24.081, 29.058)	25.996 (22.980, 29.012)	25.210 (24.095, 26.324)	26.810 (25.663, 27.958)	0.313
GST (U/g Hb)	0.354 (0.305, 0.403)	0.357 (0.297, 0.416)	0.321 (0.299, 0.343)	0.351 (0.329, 0.374)	0.515
GR (U/g Hb)	0.584 (0.525, 0.643)	0.614 (0.543, 0.686)	0.540 (0.514, 0.566)	0.558 (0.531, 0.585)	0.815
***Cognition***					
Moca Score	25.629 (24.728, 26.530)	26.467 (25.389, 27.545)	26.573 (26.168, 26.977)	26.353 (25.934, 26.772)	0.175

General linear model used. Data are presented as the mean (95% CI). For plasma Glu, TG, TC, LDL-C, and HDL-C data analysis, factors including gender, age, BMI, smoking, physical activity were adjusted. For erythrocytes antioxidant biomarkers and plasma folate, retinol, and α-tocopherol data analysis, factors including gender, age, BMI, smoking, and antioxidant supplementation were adjusted. For cognition (MoCA score) analysis, factors including gender, age, BMI, physical activity, education level, living condition, reading time per day, and antioxidant supplementation were adjusted. *p* value < 0.05 was considered as significant.

## 4. Discussion

To the best of our knowledge, this is the first population-based cross-sectional study evaluating the influence of APOE and GST polymorphism on antioxidant parameters and cognition in old Chinese adults. Our results revealed that APOE and GSTM1/T1 polymorphism was associated with lipid profile, α-tocopherol level and antioxidant parameters in plasma or erythrocyte. Besides, no association was found between APOE rs429358, rs7412 and GSTM1/T1 polymorphism and cognition in old Chinese adults.

The association of APOE genotype with plasma lipid profile was implied in the published documents [29,30,31]. Liu reported the significant relationship between APOE genotype and plasma TC and LDL-C levels in healthy women [32]. Tejedor and coworkers also observed a significant association between the APOE rs7412 as well as the rs429358 polymorphism with circulating plasma TC and TG levels [33]. In the present study, we did not observe the relationship between APOE genotypes and plasma Glu, TG and TC levels. These results are consistent with the findings of Tao’s study carried out in older Chinese. The author did not detect the association between APOE genotype and serum levels of Glu or TG [34]. Meanwhile, after including GSTT1 genotype into the data analysis, we found that the subjects with APOE rs429358 C/C and GSTT1− genotype have the highest plasma TG levels, and the statistical significance of genotype interaction was detected (Table 5). These results indicated that the deletion of GSTT1 gene will increase the plasma TG levels in the subjects with APOE rs429358 C/C genotype.

We did not observe the effect of APOE rs429358 or rs7412 polymorphism on cognition (MoCA score) in the old Chinese subjects. This result was in line with Shahar and coworker’s study [35]. In their study, the authors found that APOE genotype was not associated with mild cognitive impairment (MCI). Quintino-Santos and Blasi *et al.* also reported that APOE variation was not correlated with the overall cognitive performance as evaluated by the Mini-Mental State Examination (MMSE) test [36,37]. In addition, we did not observe the overlap of APOE and GSTM1/T1 polymorphism on the cognition among the detected older adults. Our results were consistent with Bernardini’s case-control study, in which the authors did not observe the association between the GSTM1 and GSTT1 deleted genotypes and late-onset AD (LOAD) [38]. Moreover, Spalletta reported that GSTT1 null phenotype predicted the faster onset of the AD, and the author concluded that the faster decline of cognition was independent from APOE genotype [39]. GST and APOE gene products are implicated in oxidative stress and apoptosis processes leading to beta-amyloid-mediated neurodegeneration. Therefore, further research is needed to determine the influence of APOE and GSTM1/T1 polymorphism on cognitive impairment among a larger random sample of Chinese elderly.

Vitamin E (VE) is a physiological scavenger of reactive oxygen species (ROS) produced during lipid peroxidation. VE is also capable to act as a neuroprotective and anti-inflammatory agent [40]. Shahar and coworkers reported that Vitamin E deficiency was more prevalent among APOE ε4 carriers than non-carriers among the non-MCI subjects, which indicated the role of APOE genotype in influencing serum Vitamin E concentration among elderly people with normal cognitive function. In the current study, we found that plasma VE (α-tocopherol) level was APOE rs429358 polymorphism related (Table 2). After including GSTM1 genotype into the dada analysis, we found that both APOE rs429358 and GSTM1 polymorphism affected the plasma VE level (*P_APOE rs429358 genotype_* = 0.018; *P _GSTM1genotype_* = 0.039). Subjects with APOE rs429358 C/C and GSTM1+ genotype seem to have the highest plasma α-tocopherol level (Table 4) when comparing with subjects with other genotypes. However, we did not detect the interaction between APOE rs429358 and GSTM1 polymorphism on plasma α-tocopherol level (*P_genotype interaction_* = 0.144).

The combined effects of APOE rs429358 and GSTT1 polymorphism on the plasma TG level, erythrocyte CAT and GST enzyme activities were detected (Table 5). We also observed the genotype interaction of APOE rs429358 and GSTT1 on erythrocyte CAT and GST enzyme activity (Table 5). These results indicated that the GSTT1 genotype could modify the influence of APOE rs429358 T/C + C/C genotype on erythrocytes antioxidant enzyme activities.

The plasma LDL-C level was mainly APOE rs7412 polymorphism related, and the subjects with rs7412 T/C + T/T genotype have a lower plasma LDL-C level than the subjects with C/C genotypes (Table 3). Abdollahi *et al.* [41] demonstrated in 3271 individuals (British women, 60 years and older) that the serum level of TC, HDL-C, LDL-C and TG were all associated with APOE genotype. Serum TC and LDL-C levels were lower in individuals with the APOE rs7412 T/C + T/T genotype and higher in those with the APOE rs429358 C/T + C/C genotype. Additionally, we also observed the influence of APOE rs7412 polymorphism on plasma T-AOC level. These results were in agreement with the data of Talmud and coworkers’ study [42]. After including GSTT1 genotype into the data analysis, we found that the deletion of GSTT1 gene further decreased the plasma LDL-C level in the subjects with APOE rs7412 T/C + T/T genotype, while, the subjects with APOE rs7412 C/C and GSTT1− genotype have the highest plasma LDL-C levels. In addition, we observed the significant genotype interaction of GSTM1 and APOE rs7412 on erythrocyte CAT (*P_genotype interaction_* = 0.002) and GSH-Px activity (*P_genotype interaction_* = 0.028) (Table 6). Our results indicated that GSTM1 polymorphism could strongly modify the influence of APOE rs7412 polymorphism on erythrocyte CAT and GSH-Px enzyme activities, especially on the enzyme activity of the subjects with APOE rs7412 T/C + T/T genotype.

There are some limitations in the present study. First, this is a cross-sectional study, which limits the interpretation of cause and effect. Thus, further longitudinal studies are needed to provide scientific evidence of the role of APOE rs429358, rs7412 and GST polymorphism in associating with antioxidant parameters and cognition in the elderly. Second, the study was carried out in old Chinese adults. The phenotype frequencies of APOE or GST gene in different populations show different patterns. Besides, the relationship between APOE rs429358, rs7412 and GST polymorphism with blood parameter profiles might vary depending on ethnicity and the prevailing regional environmental factors (such as lifestyle and diet pattern) [43,44,45,46,47]. Therefore, the extrapolation from the present study to others should be considered with caution. Finally, multiple tests were applied in the measuring of biomarkers and the analyzing of data in the present study. As a result, some unpredictable and uncontrollable factors might lead to the significant findings. Thus, further studies are needed to replicate and clarify the results.

## 5. Conclusions

In conclusion, plasma and erythrocytes’ antioxidant parameters levels were associated with APOE rs429358, rs7412 polymorphism. The influences of APOE rs429358 polymorphism on plasma and erythrocytes’ antioxidant parameters could be modified by GSTT1 genotype; the influences of APOE rs7412 could be modified by GSTM1 genotype.
